# Genome-wide analysis of allelic imbalance in prostate cancer using the Affymetrix 50K SNP mapping array

**DOI:** 10.1038/sj.bjc.6603476

**Published:** 2007-01-23

**Authors:** N Tørring, M Borre, K D Sørensen, C L Andersen, C Wiuf, T F Ørntoft

**Affiliations:** 1 Molecular Diagnostic Laboratory, Department of Clinical Biochemistry, Skejby Sygehus, Aarhus University Hospital, Brendstrupgaardsvej 100, DK-8200 Aarhus, Denmark; 2 Department of Urology, Skejby Sygehus, Aarhus University Hospital, Brendstrupgaardsvej 100, DK-8200 Aarhus, Denmark; 3 BiRC – Bioinformatics Research Center, University of Aarhus, Høegh-Guldbergsgade, Building 090, DK-8000 Aarhus, Denmark

**Keywords:** SNP, prostate cancer, LOH, allelic imbalance

## Abstract

Prostate cancer (PCa) is the most commonly diagnosed non-cutaneous cancer in male subjects in Western countries. The widespread use of prostate-specific antigen (PSA) has increased the detection of this cancer form in earlier stages. Moreover, it has increased the need for new diagnostic procedures to be developed for patient stratification based on risk of progression. We analysed laser-microdissected prostate tumour tissue from 43 patients with histologically verified PCa, using the new high-resolution Affymetrix Mapping 50K single-nucleotide polymorphism array. The results showed six major loss of heterozygosity regions at chromosomes 6q14–16, 8p23–11, 10q23, 13q13–21 and 16q21–24 and a novel region at chromosome 21q22.2, all of which reveal concomitant copy number loss. Tumour development was further characterised by numerous novel genomic regions almost exclusively showing copy number loss. However, tumour progression towards a metastatic stage, as well as poor differentiation, was identified by specific patterns of copy number gains of genomic regions located at chromosomes 8q, 1q, 3q and 7q. Androgen ablation therapy was further characterised by copy gain at chromosomes 2p and 10q. In conclusion, patterns of allelic imbalance were discovered in PCa, consisting allelic loss as an early event in tumour development, and distinct patterns of allelic amplification related to tumour progression and poor differentiation.

Prostate cancer (PCa) is one of the most commonly diagnosed male malignancies in Western countries, and a leading cause of cancer-related death (Landis *et al*, 1999).

The widespread use of prostate-specific antigen (PSA) in the diagnosis of PCa has increased the detection of this cancer in earlier stages. Although this development has increased the possibilities to cure patients with PCa, the morbidity of the disease has equally increased and has pushed the demand for stratification of treatment for patients with PCa. Because PCa often progresses slowly, a subset of patients with early-stage disease may be candidates for watchful waiting rather than surgical treatment. Currently, it is impossible to discriminate between latent and aggressive cancers at an early state of disease. Furthermore, clinicians cannot predict how slowly or rapidly a cancer will grow, or whether a cancer has the potential to metastasise. Development and progression of PCa from a localised disease to hormone-refractory and metastatic stage is driven by a multistep process with accumulation of multiple genetic and epigenetic changes in specific genes (Quinn *et al*, 2005). Altered transcript levels in cancer genomes are often related to copy number changes (Pinkel and Albertson, 2005), and genome-wide detection of allelic imbalance in cancer tissue by polymorphic genetic markers has become an important technique to identify genetic events involved in the aetiology and progression of human cancers (Balmain *et al*, 2003). Until recently, the technology represented by PCR-based determination of microsatellites only allowed a modest number of polymorphic markers to be used, which limited the resolution of the technique. With the development of comparative genomic hybridisation (CGH) arrays using more than 30 000 BAC clones spanning the human genome (Ishkanian *et al*, 2004), and high-density single-nucleotide polymorphism (SNP) microarrays designed to genotype more than 100 000 SNPs in the human genome DNA (Matsuzaki *et al*, 2004), the resolution of the whole genome scanning technique has increased considerably and allowed accurate and reproducible determination of copy number changes in the cancer genome (Goyama *et al*, 2004; Nannya *et al*, 2005). The SNP array offers the possibility to analyse LOH and generate accurate copy number simultaneously in a high-throughput and high-resolution genome-wide manner, thus making it possible to distinguish between LOH regions with underlying hemizygous deletions and those with copy-neutral events (Zhao *et al*, 2004).

The identification of genomic areas showing copy loss is highly dependent on sampling of pure tumour DNA without contamination of normal epithelium, stroma and/or inflammatory cells (Zhao *et al*, 2004). We therefore performed laser microdissection to obtain pure samples of prostate adenocarcinoma, and Affymetrix SNP arrays were applied to 43 phenotypically well-characterised PCas spanning from localised PCa to metastatic disease with and without previous androgen deprivation. In the present study, we report results based on an array with more than 50 000 SNPs, and this remarkable increase in resolution defined distinct patterns of chromosomal loss confined to PCa development and patterns of chromosomal gains confined to PCa progression and poor differention (Gleason score ⩾8).

## MATERIALS AND METHODS

### Sample collection and clinical data

Samples of prostate adenocarcinoma were selected from series of patients with PCa who underwent surgery for their disease at the Department of Urology, Skejby Sygehus during the periods 1994–1996 and 2003–2004. The tissue was isolated as either chips from transurethral resection of the prostate (TURP) from patients with metastatic PCa who received palliative treatment or as four needle biopsies (gauge 12–14), two from each lobe obtained during surgery for radical prostatectomy, from patients with histologically verified PCa. The tissue was stored either as fresh frozen or ‘Tissue-Tek’-embedded tissue and stored at −80°C until examination. All cases were reviewed by a qualified uropathologist. The sections were examined and areas of adenocarcinoma were identified. In approximately 40% of the patients, the biopsies did not contain adenocarcinoma tissue, and these patients were therefore excluded from the study. Blood samples were obtained from patients with PCa and frozen as a source of normal germline DNA. In total, 87 samples were analysed: 43 samples of DNA from PCa tissue and 44 samples of germline DNA from blood samples. Of these samples, 39 were matched germline and cancer DNA. Clinicopathological data were obtained from the medical records. The study protocol was approved by the ethical committee in the Aarhus Council.

### Laser microdissection of prostate adenocarcinoma

In brief, 5 *μ*m thin sections of prostate tissue were cut on a cryostat and placed on PALM® membrane slides. After haematoxylin staining (Sigma Aldrich-Denmark, Brondby, Denmark) for 3 min, the samples were rinsed in H_2_O and stained in eosin (Sigma Aldrich) for 10 s. After rinsing the slides in H_2_O, they were dehydrated in increasing concentrations of ethanol and air-dried for 10 min. Prostate adenocarcinoma cells were isolated by laser microdissection using the PALM system (PALM Microlaser Technologies AG, Bernried, Germany). An area of approximately 10–30 mm^2^ was dissected from each patient and isolated in 300 *μ*l of lysis buffer (Gentra Systems Inc., Minneapolis, MN, USA).

### DNA extraction

DNA was extracted using of the PureGene DNA extraction kit (Gentra Systems Inc., MN, USA) (www.gentra.com). A 10 *μ*l volume of Proteinase K (5 mg ml^−1^) was added to the lysis buffer. Protein precipitate solution was added and the sample was centrifuged. The supernatant was isolated and 1 *μ*l of linear polyacrylamid (10 *μ*g *μ*l^−1^) and 300 *μ*l of isopropanol were added. The sample was centrifuged, the supernatant was discharged and the DNA pellet was washed in 70% ethanol. The supernatant was discharged and the dried pellet was rehydrated in 15 *μ*l of rehydration solution. On average 500 ng to 1 *μ*g DNA was obtained from each sample.

Spectophotometric absorbance was measured at 260 and 280 nm. Ratios (OD_260_/OD_280_) of 1.4±0.2 (mean±standard deviation (s.d.)) were obtained for DNA extracted from laser-microdissected PCa tissue and ratios of 1.8±0.1 were obtained for blood DNA.

### GeneChip® mapping 50K array

Array experiments were performed according to the Affymetrix GeneChip Mapping 50K array standard protocol (Affymetrix Inc., Santa Clara, CA, USA). The Mapping 50K system consists of two 8-*μ*m arrays each designed to hybridise labelled PCR fragments from *Xba*I- and *Hin*dIII-cleaved DNA. To reduce the costs of the project, only the array containing probes for *Xba*I-cleaved DNA detecting 58 960 SNPs was used. A 250 ng measure of DNA was used for the Mapping 50K array. We obtained a call rate of 92.9±7% (mean±s.d.) in the tissue samples and 96.1±4.5% in the germline samples, thus comparable to the call rates of the previous generation of SNP array Mapping 10K from Affymetrix (Koed *et al*, 2005).

### Data analysis

The physical position of all SNPs (*n*=58 960). on the Mapping 100K array was mapped according to the May 2004 genome assembly (hg17) at http://genome.ucsc.edu/

Single-nucleotide polymorphisms that did not map or mapped to more than one position in the genome assembly were excluded from the analysis, leaving 57 429 SNPs.

DNA analysis software (GDAS) 3.0.2 Patch Software (Affymetrix Inc., CA, USA) was used to generate genotype calls. Genotypes and probe intensities derived from germline and cancer DNA were loaded into the software package dChip (http://www.dchip.org/) (Lin *et al*, 2004), which was used for LOH analysis: the probe intensities were normalised and a single signal value (the observed signal) for each SNP in each array was obtained. Loss of heterozygosity calls were obtained for the 39 samples for which matched tumour and germline DNA exist.

### Validation of SNP genotypes

The accuracy of SNP genotype calls generated by the Affymetrix GDAS 3.0.2 software was evaluated by comparison with genotyping obtained independently by a single-base extension method using the ABI PRISM® SNaPshot™ Multiplex kit (Applied Biosystems, Foster City, CA, USA) as previously described (Gaustadnes *et al*, 2006). A list of all the primers used for PCR amplification and single-base extension SNP genotyping is shown in Supplementary Information (Table 1). The DNA samples were similar to the samples used for SNP Chip analysis.

### Quantitative PCR

Real-time PCR was performed on an ABI PRISM® 7000 Sequence Detection System (Applied Biosystems, Foster City, CA, USA), using the SYBR® GREEN PCR Master Mix (Applied Biosystems). Quantification of four different target genes (MAP3K7, PPP3CC; SGCZ and CSMD1) was based on standard curves constructed from four-fold serially diluted normal genomic DNA samples. The copy number of each target was determined relative to a reference Line-1 repetitive element, with a method previously described by others in detail (Zhao *et al*, 2004). A complete list of primer sequences is given in Supplementary Information (Table 2). Mann–Whitney non-parametric test was used to determine the difference in copy number between groups. GrapaPad Prism4 (San Diego, CA, USA) was used as statistical software.

### Extraction of weighted signal intensities

After normalisation and extraction of signal values for the 87 arrays (43 tumours and 44 germline samples), as described in Data analysis, the data were further normalised SNP-wise to allow comparison between different SNPs. Signal values are not directly comparable because probe sets representing different SNPs on the array have different physical properties. Therefore, the data were normalised SNP-wise using the mean and s.d. of the germline samples, that is, *z*_*ij*_=(*x*_*ij*_−mean_*j*_)/s.d._*j*_, where *x*_*ij*_ is the observed signal of SNP *j* in sample *i* (germ line or tumour), and mean_*j*_ and s.d._*j*_ are the mean and standard deviation of SNP *j* for all 44 germline samples. As a consequence, the mean and s.d. of *z*_*ij*_ is 0 and 1, respectively, for all the SNPs in the germline samples. To further reduce the noise level in the signal values, we calculate the average of *M*=9 SNPs weighted by genomic distance; that is, *a*_*ij*_=Σ*z*_*i*(*j*+l)_ exp(−*d*_*j*(*j*+*l*)_)/Σexp(−d_*j*(*j*+*l*)_), where the sum (Σ) is over the four neighbouring SNPs on both sides of SNP *j* (*l*=−4,−3,…,0,…,3,4) and d_*j*(*j*+*l*)_ is the genomic distance between SNP *j* and *j*+*l*. *a*_*ij*_ is referred to as the weighted signal intensity – in the following referred to as signal intensities.

### Mapping of genomic regions commonly showing copy number alterations

Genomic regions commonly showing copy number alterations were identified as segments of consecutive SNPs, for which the average weighted signal intensity (over all tumour samples) was significantly different from the average weighted signal intensity of the germline samples (*P*⩽0.01). Only regions of ⩾40 consecutive SNPs, each with *P*⩽0.01, were reported. The null distribution of germline samples was obtained by randomly permuting the array labels (tumour and germ line) 10 000 times and computing the difference in averages for each permutation.

### Genomic differences between tumour subgroups

For different subgroups of tumours that were defined by metastasis status, tumour stage or androgen deprivation status, the differences were identified based on weighted signal intensities.

For each subgroup, the average weighted signal intensity was calculated and plotted. The significance of the difference of the group means was calculated using a permutation test. Group labels were randomly shuffled 10 000 times, the difference recalculated and the number of times a value larger/smaller than the observed group difference was counted. Note: the group labels were reassigned sample-wise and not SNP-wise, such that the dependencies between SNPs were maintained. To assess the significance of the observed pattern of differences, it was evaluated how often the observed segments of SNPs were found in the distribution obtained by permuting group labels. Segments with *P*⩽0.01 were reported. At this level, it is expected that less than one chromosome (22 × 0.01=0.22) show a significant segment of SNPs. An upper bound to the false discovery rate of chromosomes with reported significant segments is thus 1/no. of chromosomes with significant segments.

## RESULTS

### Comparison of genotype calls

Forty-three microdissected tissue samples of PCa and 44 samples of germline DNA from patients with PCa (Table 3; Supplementary Information) were analysed for allelic imbalance and copy number alterations using the Mapping 50K SNP chip from Affymetrix.

In order to validate the genotype calls from the Affymetrix GDAS software, we compared the genotype call from five individual SNPs in a total of 114 alleles in five different genes from Affymetrix GDAS software to the ABI SNaPshot single base extension method (Gaustadnes *et al*, 2006). We found a 98% concordance between the genotype calls in Affymetrix GDAS software, and by SnaPshot® single base extension. Two alleles were inconclusive by SNaPshot SBE. This indicates a high degree of consistency of the genotype call derived from the Affymetrix GDAS software. A list of genes is shown in Supplementary Table 4.

### Genomic regions commonly showing LOH

**Figure 1:**
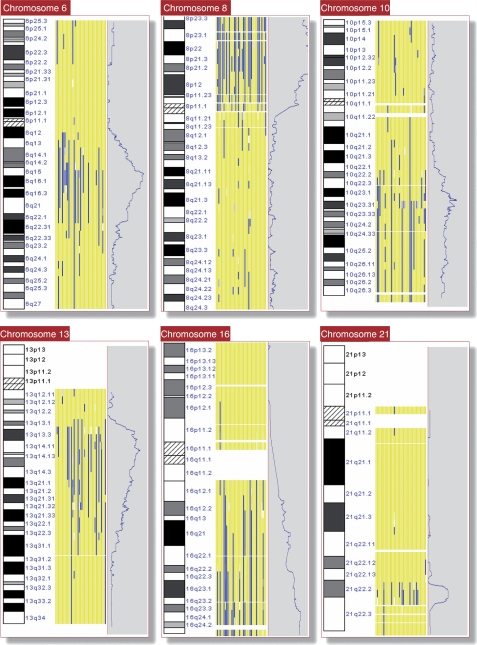
Loss of heterozygosity in chromosomes 6, 8, 10, 13, 16 and 21 in microdissected prostate cancer tissue as determined by dChip. Loss of heterozygosity regions (blue), retained regions (yellow) and uninformative (white) covering the genome in 39 individual samples of matched tumour and germ line. Each column represents one tumour/germline pair. Additionally, along the right-hand side of each figure within the grey shaded box is the average LOH score for the 39 samples. Cytoband for the individual chromosomes is shown on the left-hand side. Chromosomes 1–22 are shown in Supplementary Figure 4.

The conversion of a heterozygous SNP in normal blood cells to a homozygous SNP in tumour tissue indicates the loss of one allele (LOH) in the tumour. The overall pattern of LOH in the 39 samples of prostate adenocarcinoma, as determined by dChip software, is shown in Figure 1, Table 1 and Figure 4 (Supplementary Information). The genomic areas showing frequent LOH were defined as areas showing LOH in more than 30% of samples (⩾12 of 39 cases). The regions that range in size from 1.5 to 22.1 Mb include positions at 6q, 8p, 10q, 13q and 16q. The highest percentage of LOH (>60%) was confined to a 1.5-Mb region at chromosome 8p21.3. The high-resolution SNP array also provides an excellent tool for discovering novel alterations. One novel region of frequent recurrent deletion, at 21q22.2, was of particular interest, because *ERG* and *TMPRSS2*, both of which are located at this region, were recently found to be involved in common gene fusion events in PCa (Tomlins *et al*, 2005). This novel LOH region covering 2.9 Mb at chromosome 21q22.2 showed LOH in 12 of the samples.

### Genomic regions commonly showing copy number alterations

Regions commonly showing copy number alterations were identified by comparing the signal intensities from the Mapping 50K array in the tumour group (*n*=43) with the intensities of the 44 germline samples. Using a cutoff of ⩾40 consecutive SNPs each displaying significant difference (*P*⩽0.01) between prostate tumour and germline samples, we identified 73 genomic regions. The size of the regions extended from 0.5 to 9.6 Mb (Table 2). Out of the 73 regions identified, only two regions at chromosomes 2q24 and 20q13.3 showed a gain. Multiple regions at chromosomes 2, 5, 6, 8, 10, 13, 16 and 18 sharing losses are not necessarily independent regions of altered DNA copy changes, but could be subfractions of larger regions.

### Comparison of LOH and copy number alterations in tumour DNA

**Figure 2:**
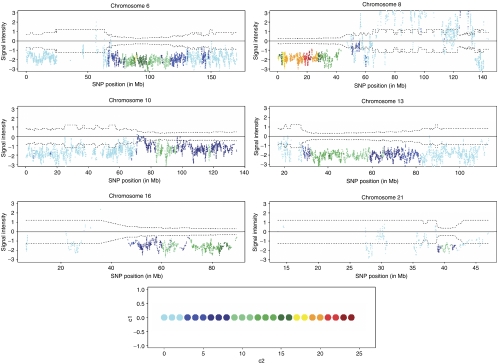
Correlation between LOH and genomic copy number alterations in SNPs showing LOH. The signal intensity value for a particular SNP was calculated for all tumours with LOH in that particular SNP and plotted with a colour that indicates the number of tumours with LOH. Dotted lines correspond to a significance level of 1%. The widths of these vary because some SNPs experience more LOH than others. Single-nucleotide polymorphisms with signal intensities outside the 1% significance level threshold are considered to represent genomic copy numbers different from 2 (if positive >2 and if negative <2). Areas for which LOH and copy number reductions seem to be positively correlated include 6q, 8p, 10q, 13q, 16q and 21q. Inserted colour code shows number of samples with LOH.

Recently it has been shown that LOH regions in the cancer genome often show two copies, despite the loss of one allele (Cleton-Jansen *et al*, 2004). We therefore tested the correlation between LOH and signal intensities for all samples showing LOH. As shown in Figure 2, the major LOH regions at chromosomes 6, 8, 10, 13, 16 and 21 all showed a positive correlation between LOH and significantly decreased weighted signal intensities. This indicates that loss of one allele in PCa correlates to a decrease in copy number. The LOH/signal intensity plot for all chromosomes is shown in Supplementary Figure 1. We further tested the same correlation for the subgroups of localised and metastatic PCa to determine whether mitotic recombination would be found preferentially in advanced tumours as compared with early stages as shown in advanced stages of breast cancer (Cleton-Jansen *et al*, 2004), but no difference in the individual groups was observed (data not shown).

To validate the results, we determined the copy number score by real-time PCR for samples showing LOH in four different genes in LOH regions at chromosomes 8p and 6q.

The results showed a copy number of 1.01±0.2 (mean±s.d.) (*n*=30) in tumour DNA as compared with 2.1±0.2 (*n*=28) in germline DNA. This confirms that copy number loss is evident in samples showing LOH.

### Genomic regions of allelic imbalance in subgroups of tumours showing poor differentiation and in metastatic disease

In order to identify genomic regions showing significant alterations in copy number changes in subgroups of prostate tumours, we compared the signal intensities from the Mapping 50K chips. Genomic regions defined as regions containing consecutive SNPs each showed a significant difference between groups of *P*⩽0.01.

Two of the samples could not be classified concerning metastasis status and were excluded. Samples from metastatic PCa (*n*=22) as compared with localised PCa (*n*=19) were characterised by the almost exclusively increased copy number throughout the genome, including large regions at chromosomes 1q, 8q, 9q, 11q, 12q and 17q, as listed in Supplementary Table 5A. Out of the 22 regions identified, only one 0.4-Mb region at chromosome 4p showed decreased copy number in metastatic PCa as compared with localised disease.

**Figure 3:**
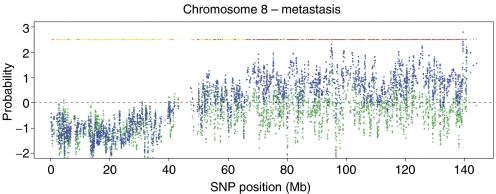
Signal intensities in chromosome 8. Genomic differences between tumour subgroups were identified based on signal intensities. For each group, the signal intensity was calculated and plotted. The significance of the difference of the group means was calculated using a permutation test. Group of metastatic disease (blue) and localised disease (green). Significance is indicated on top: *P*⩽0.01 (brown), 0.01⩽*P*⩽0.02 (red), 0.02⩽*P*⩽0.05 (orange).

As illustrated in Figure 3, the genomic changes at chromosome 8q are characterised by gain over large regions exclusively in samples from metastatic PCa, whereas loss of 8p is shared between samples from localised and metastatic disease. Genomic changes in all chromosomes in samples from metastatic *vs* localised PCa are shown in Supplementary Figure 2. The only major region showing a tendency towards lower signal intensities in samples of metastatic disease as compared with localised disease was the LOH region at chromosome 6q14–16. Lowering the cutoff *P*-value of the individual SNPs from 0.01 to 0.02 showed that the copy loss of 6q14–16 was statistically more common in metastatic disease (*P*<0.05), suggesting a higher frequency of copy loss in the samples of metastatic disease as compared with organ-confined disease (data now shown). The group of samples from patients with metastatic cancers was further divided into groups from patients who were given (*n*=7) or not given (*n*=15) androgen deprivation treatment. Genomic regions at chromosomes 2p21, 2p16 and 10q21 showed significantly increased copy number in the samples from patients who received androgen ablation therapy (Table 5B (suppl)+Figure 3 (suppl)).

Similar patterns of exclusively increased copy numbers were seen from samples displaying a Gleason score ⩾8 (*n*=23) when compared with a Gleason score ⩽7 (*n*=19). Increased copy numbers at positions 1q32, 7q32, 8q22 and 8q24.1 were shared between samples from patients with metastatic disease and poorly differentiated tumour (Gleason score ⩾8) (Table 5C (suppl)).

We further compared the signal intensities for samples from patients with organ-confined (T2a–c) *vs* locally invasive tumours (T3a+b), but no regions reached the cutoff values indicated above.

## DISCUSSION

Using the new Mapping 50K SNParray from Affymetrix, we performed a high-resolution global scale screening for allelic imbalance in 43 laser-microdissected samples of PCa. The results showed a set of genomic alterations characteristic to PCa, consisting of LOH and concomitant copy loss in large regions at chromosomes 6q, 8p, 10q, 13q and 16q. Most of these affected regions have previously been associated with PCa (Joos *et al*, 1995; Cooney *et al*, 1996; Elo *et al*, 1997; Feilotter *et al*, 1998; Hyytinen *et al*, 1999; Lin *et al*, 2004). However, the increase in resolution using high-density SNP arrays combined with laser microdissection enabled us to identify regions that have not previously been related to this disease, including a 2.9-Mb region showing LOH at chromosome 21q22.2 in 30% of the samples. Loss of 21q22 has previously been reported in gastric cancer and NSCLC (Park *et al*, 2000; Tseng *et al*, 2005), but the LOH region we discovered in PCa at 21q22.2 is more distant and therefore previously unknown in relation to carcinomas. Recently, Tomlins *et al* (2005) identified a highly frequent recurrent gene fusion in PCa, including ERG and TMPRSS at chromosome 21q22, and was recently confirmed by Yoshimoto *et al* (2006). Interestingly, ERG and TMPRSS genes are both located exactly at the outer borders of this new LOH region at 21q22, and support the results showing fusion between these two genes. During revision of this manuscript, Liu *et al* (2006) reported a similar common region of deletion between the ERG and TMPRSS2 genes on chromosome 21, presumably related to the recently identified fusion transcripts from these two genes in 25% of samples of PCa.

Using the signal intensities to determine the significant copy number changes between tumour and germline samples, additional regions were identified at chromosomes 2–5, 9, 11, 14, 15, 18 and 20. With a cutoff *P*-value of 0.01 for ⩾40 consecutive SNPs, 73 regions ranging in size from approximately 0.5to 10 Mb were identified. All regions except for two showed copy loss in the tumour samples, indicating that development of PCa is characterised almost exclusively by the loss of specific genomic regions. The majority of the smaller regions represent genomic regions that have not previously been related to PCa. Combined global analysis of LOH and genomic copy number data showed that LOH in PCa correlates positively to copy loss. Our data showed no signs of uniparental disomy in LOH regions, as otherwise recently reported to be a common mechanism in advanced breast cancer (Murthy *et al*, 2002; Cleton-Jansen *et al*, 2004), acute myeloid leukaemia (Raghavan *et al*, 2005), medullablastoma (Langdon *et al*, 2006) and basal cell carcinomas (Teh *et al*, 2005).

Loss of heterozygosity and concomitant copy loss at chromosomes 8p, 10q, 13q,16q and 21q were found with an equal frequency in both localised and metastatic tumours, and were not associated with tumour stage or grade. The only LOH region that showed a tendency towards higher degree of copy loss in metastatic and low differentiated tumours was chromosome 6q16.

Previous reports have indicated that early-stage organ-confined prostate tumours did not display chromosome-level imbalances, and that balanced cytogenetic and epigenetic changes could be responsible for tumour development (Fu *et al*, 2000; Chu *et al*, 2003). A combination of microdissection of tumour tissue to maximise the yield of neoplastic tissue and a new high-resolution methodology enabled us to show that LOH and copy number loss have similar frequency in organ-confined and metastatic tumours, indicating that LOH is an early event in PCa tumour development. Recently, it has been shown that LOH at chromosomes 6q, 8p and 10q occurs in high-grade PIN lesions (Wang *et al*, 2001; Wang and Lai, 2004a, 2004b), which is now accepted as the most likely preinvasive stage of prostate adenocarcinoma. Ribeiro *et al* (2006) recently hypothesised that LOH at 8p and 13q are distinct initiation events in the carcinogenesis of PCa. Our data do not support this theory as LOH of the two regions occurs randomly in our samples. On average, we observed LOH at 3–4 regions in each sample, and suggest that accumulation of specific genomic losses occurs as independent events. Although LOH is a common event in PCa, approximately 13% of our samples (five out of 39) reveal no sign of LOH, and were thus chromosomally stable. This number is lower than 31% of samples reported as chromosomally stable using the CGH technique (Teixeira *et al*, 2004).

We then investigated the genomic copy number changes in relation to tumour stage and Gleason grade. Interestingly, the analysis revealed 31 genomic regions at chromosomes 1, 4, 5, 8–12, 14, 17 and 19–22, all except one showing significantly increased copy number in tumours from patients with metastatic disease as compared with localised disease. The regions identified at chromosome 8q are similar to the regions recently identified by high-resolution CGH (van Duin *et al*, 2005), showing amplification of 8q harbouring genes like C-MYC and EIF3S3, and gain of 17q25 in advanced cases of PCa (Ribeiro *et al*, 2006). The region at 5p13 showing copy gain harbours the F-box protein SKP2, which is upregulated in advanced PCa (Dhanasekaran *et al*, 2001) and was found to induce PCa in a transgenic mouse model (Shim *et al*, 2003). Copy gain at chromosome 11q13 was shown to predict postoperative recurrence independent of stage and grade, but a specific biomarker has not been identified at this region (Paris *et al*, 2004).

The copy number changes in relation to tumour grade showed a similar pattern of exclusively upregulation in numerous regions at chromosomes 1q, 3q, 7q, 8q and 14p in samples displaying high Gleason grade. There was a considerable overlap to regions identified also in samples from patients with metastatic PCa at chromosomes 1q, 7q and 8q. The majority of the regions showing increase in copy number were novel, and these areas should be examined for potential oncogenes and their ability to predict the course of disease in a larger study with long-term follow-up.

Based on the results, we propose a genetic pathway of prostate carcinogenesis with distinct initiation events, namely loss of chromosomes 8p, 13q, 16q and 21q. As tumours progress, prostate carcinomas display increased genomic complexity, leading to genomic imbalances including loss of 6q and gain at chromosomes 8q, 1q, 7q and 3q, and this enables the tumour to metastasise. Prospective studies with sufficiently long follow-up time, allowing for disease survival as the clinical endpoint, are necessary to determine the clinical use of allelic imbalance as a prognostic marker.

In conclusion, our analysis revealed a characteristic pattern of genomic imbalances in adenocarcinoma tissue from patients with PCa and identified genomic regions highly associated with tumour initiation, metastasis and high-grade disease. Our results indicate that allelic loss is an early event in prostate tumour development, and that allelic amplification is exclusively confined to tumours that progress. Future studies should evaluate the use of a specific pattern of genomic allelic imbalances as a prognostic marker in PCa.

## Figures and Tables

**Table 1:**
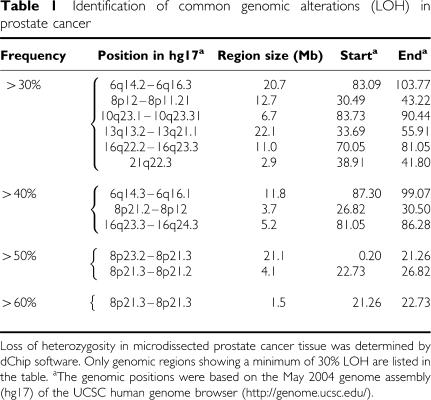
Identification of common genomic alterations (LOH) in prostate cancer

**Table 2:** Genomic regions with altered signal intensities

**Chromosomes**	**Start^a^**	**End^a^**	**SNPs in region**	**Region size (Mb)**	**Gain/loss**
1	70.31	72.72	77	2.41	Loss
2	40.76	42.08	63	1.32	Loss
2	49.73	50.73	51	1.00	Loss
2	58.12	60.63	64	2.51	Loss
2	67.05	69.07	70	2.02	Loss
2	81.78	82.95	43	1.18	Loss
2	167.63	168.44	44	0.81	Gain
3	75.95	78.11	42	2.17	Loss
4	74.14	75.72	41	1.59	Loss
4	101.42	103.12	42	1.70	Loss
4	142.78	144.48	57	1.70	Loss
4	157.13	158.67	50	1.54	Loss
5	44.28	51.99	65	7.71	Loss
5	88.12	89.87	40	1.75	Loss
5	90.12	92.31	43	2.19	Loss
5	98.57	100.62	48	2.06	Loss
5	100.69	102.38	43	1.68	Loss
5	113.51	115.68	80	2.17	Loss
6	69.21	70.54	54	1.33	Loss
6	75.47	78.16	50	2.68	Loss
6	80.96	82.84	57	1.88	Loss
6	84.03	85.89	66	1.86	Loss
6	87.45	89.36	49	1.92	Loss
6	89.92	94.20	132	4.28	Loss
6	97.38	99.27	46	1.90	Loss
6	99.39	101.86	73	2.47	Loss
6	110.16	113.42	59	3.26	Loss
6	124.66	126.15	55	1.48	Loss
8	0.18	2.76	41	2.58	Loss
8	2.90	4.28	75	1.37	Loss
8	4.47	4.95	54	0.47	Loss
8	4.95	13.61	225	8.66	Loss
8	13.61	23.19	345	9.58	Loss
8	23.42	26.58	78	3.16	Loss
8	26.58	28.16	58	1.58	Loss
8	28.36	30.84	58	2.49	Loss
9	30.09	32.01	44	1.92	Loss
10	57.71	59.11	45	1.40	Loss
10	85.45	86.83	52	1.37	Loss
10	86.94	90.33	67	3.39	Loss
10	91.63	93.03	46	1.40	Loss
10	106.09	109.86	115	3.77	Loss
10	110.09	112.14	42	2.04	Loss
11	113.53	115.20	50	1.67	Loss
13	32.84	36.54	123	3.70	Loss
13	40.07	44.20	122	4.13	Loss
13	44.68	46.13	55	1.45	Loss
13	48.79	51.40	42	2.61	Loss
13	51.59	55.83	103	4.24	Loss
13	59.02	60.10	53	1.07	Loss
13	61.96	63.70	42	1.74	Loss
13	66.67	67.98	56	1.31	Loss
13	82.86	84.22	57	1.37	Loss
13	103.27	104.28	45	1.02	Loss
14	25.10	27.56	55	2.47	Loss
15	47.67	49.27	48	1.59	Loss
16	51.69	53.25	49	1.56	Loss
16	61.10	63.24	61	2.14	Loss
16	72.85	76.17	56	3.32	Loss
16	76.19	78.12	60	1.92	Loss
16	79.68	82.40	64	2.72	Loss
16	82.48	83.24	55	0.75	Loss
16	83.25	85.62	42	2.36	Loss
18	24.47	26.26	54	1.79	Loss
18	28.67	30.80	49	2.13	Loss
18	34.40	36.59	66	2.19	Loss
18	47.67	49.11	52	1.44	Loss
18	50.65	53.59	70	2.93	Loss
18	54.14	57.06	81	2.92	Loss
18	62.51	64.11	64	1.60	Loss
18	66.15	68.29	73	2.14	Loss
20	54.18	55.45	40	1.27	Gain
21	21.83	22.70	40	0.87	Loss

Listed are regions with ⩾40 consecutive SNPs each displaying significant difference (*P*<0.01) in the signal intensity of the prostate tumour samples compared with germline samples. Multiple regions at chromosomes 2, 5, 6, 8, 10, 13. 16 and 18 are not necessarily independent regions of altered DNA copy changes, but can be parts of larger regions. Median intermarkerdistance is 17 kb.

a The genomic positions were based on the May 2004 genome assembly (hg17) of the UCSC human genome browser (http://genome.ucsc.edu/).

## Supplementary Material

Supplementary data Table 5

Figure 1 Supplement/1

Figure 1 Supplement/2

Figure 2 Supplement/1

Figure 2 Supplement/2
