# Supplementary material for: Genome-wide analysis of allelic imbalance in prostate cancer using the Affymetrix 50K SNP mapping array
Source: Br J Cancer. 2007 Jan 23;96(3):499–506. doi: 10.1038/sj.bjc.6603476 (PMC2360016; doi:10.1038/sj.bjc.6603476)
Supplement: Supplementary data Table 1 [file 6603476x1.doc]

**Table 1 Supplementary materials**

**SNaPShot single base extension primer sequences**

| **SNP** ^a^ | **Forward PCR primer** | **Reverse PCR primer** | **SBE primer** |
| --- | --- | --- | --- |
| **SERPINB5** |  |  |  |
| rs1509479 | TATGGCCTGGGACAATGTTT | CAATGGGTACCGTAGTGGTG | GTCACAAATTAGTTTTGTTGGCTGGGCTG |
| rs1858142 ^b^ | GAGTGGATGGAGAGCTGTGG | GGAGCAGGGAAGAAAGGAAG | GGATGATATATAAACAAGGGTGGATTCAGG |
| rs11873010 ^b^ | GCGCAACTCCACTATCCCTA- | TGGTCCCAGACAGTGCATAA | GGAACCAGTTGCATATACTGAGAAGAGC |
| rs1509478 ^b^ | AGACAGGAGGCCAGGTGTTA | ATGTCCCAGGAGACAACCTG | AAGAAAAGGCCTGGGCAGTGCTTGGA |
| rs2000714 ^b^ | GGGTTTCTTTCTGGCTATGG | CTTTCGCTGGCCTTTCTCTA | GGGACTTGAAGCAGCTTCACTCCTG |
| **MAP3K7** |  |  |  |
| rs205351 | GCTGCAGTAGTCTGGAGGAAAT | GGGAGGGCTTTATAGAATGCTT | TAGTATATTTGGTTCAAGAGCCGGTCG |
| rs157688 ^b^ | TGAAAATTTCTTACGCAAAGCA | CAAAGTATCTGCTGGACAAAAATG | AAGCCTAGGCCTTAAAGGTGTGTGC |
| **LTBP4** |  |  |  |
| rs1864074 | GGGGCTTGAAACAGGTATATGA | AGACAGTGCGGGCTTAGAAAT | ACTTTTGTCTTATGAAGAGATTTTGGCGTA |
| **PPP3CC** |  |  |  |
| rs1116085 | TTAAGAAGGTCCCAATTTCTCCA | AAGCATCCCAGTAGCCAAAATAG | CCAATTTCTCCACATTCTCATCAACACT |
| rs2449340 ^b^ | CGGATTAAAGGCATTTTGAGAG | TCAGCCAGTTCTTGTTTTTCAA | ACCTGCAGTTTCAATACTACATTCCAATT |
| rs1879793 ^b^ | TCCAAATCAGAAGCAAAAACCT | AAGGTTGCAGAATTTCTTGAGTG | TTAGGGGAGAGGTGTTTGGGAC |
| **SCAM-1** |  |  |  |
| rs2449346 ^b^ | TCAAGGACTCACTGAATGGATG | CTTCTCCCAGGCTCTAATGAGT | CCTTAAGTCCCTCTCTGATTTCCC |

^a^ All analyzed SNPs are intragenic.

^b^ SNP not represented on XbaI 50K SNP array.
