# Supplementary material for: Genome-wide analysis of allelic imbalance in prostate cancer using the Affymetrix 50K SNP mapping array
Source: Br J Cancer. 2007 Jan 23;96(3):499–506. doi: 10.1038/sj.bjc.6603476 (PMC2360016; doi:10.1038/sj.bjc.6603476)
Supplement: Supplementary data Table 2 [file 6603476x2.doc]

**Table 2 Supplementary materials**

**Q-PCR primer sequences**

| **Gene** | **Gene ID** | **Forward primer** | **Reverse primer** |
| --- | --- | --- | --- |
| MAP3K7 | 6885 | GAATTAGCGCTTTGGGTTGC | TTTCTTTCGCAGTGCTGCAT |
| PPP3CC | 5533 | CCTGCAGTTTGTGAATTTTTGC | AAAGACTCTGCAGCGATATTTGG |
| SGCZ | 137868 | GATTACCATTGGGGCTGAAAAG | AAGAAGCTCCTTGTGCAGTTGT |
| CSMD1 | 64478 | GGTGTTGCCTCATACGATCAAG | AAATGTGATATTGAAGCCCTCGT |
| LINE-1^a^ | - | AAAGCCGCTCAACTACATGG | TGCTTTGAATGCGTCCCAGAG |

^a^ Primer sequences as described in (ref)

All reactions were done in triplicate using the following PCR conditions: 50° C for 2 min; 95° C for 10 min; 40 cycles of 95° C for 15 sec and 60° C for 1 min.

Primers for Line-1 were as described in (11), whereas we used the Primer3 software (<http://frodo.wi.mit.edu/cgi-bin/primer3/primer3_www.cgi>) to design new primer sets spanning a 100-150-bp non-repetitive region of each of the target genes. The specificity of each primer set was validated by electronic PCR as well as BLAST searches against the complete human genome ([http://genome.ucsc.edu](http://genome.ucsc.edu/)). Furthermore, melting analysis of real-time PCR end-products along with agarose gel electrophoresis confirmed each primer set to generate only a single amplicon of the expected size.
