# Supplementary material for: Genome-wide analysis of allelic imbalance in prostate cancer using the Affymetrix 50K SNP mapping array
Source: Br J Cancer. 2007 Jan 23;96(3):499–506. doi: 10.1038/sj.bjc.6603476 (PMC2360016; doi:10.1038/sj.bjc.6603476)
Supplement: Supplementary data Table 3 [file 6603476x3.doc]

**Tabel 3 Supplementary Materials**

**Clinical and histopathological classification of prostate adenocarcinoma samples**

Sample No. Gleason score T-class M-class Androgen deprivation

1 7 2a 0 0

2 6 2b 0 0

3 7 2b 0 0

4 7 2b 0 0

5 7 2b 0 0

6 7 2b 0 0

7 7 2b 0 0

8 9 2b 0 0

9 7 2c 0 0

10 7 3a 0 0

11 7 3a 0 0

12 7 3a 0 0

13 7 3a 0 0

14 7 3a 0 0

15 7 3a 0 0

16 7 3a 0 0

17 9 3a 0 0

18 7 3b 0 0

19 8 3b 0 0

20 7 >2* 1 0

21 7 >2* x 0

22 8 >2* 1 0

23 9 >2* x 0

24 9 >2* 1 0

25 9 >2* 1 0

26 10 >2* 1 0

27 8 >2* 1 1

28 8 4 1 0

29 9 4 1 0

30 7 4 1 0

31 8 4 1 0

32 9 4 1 0

33 7 4 1 0

34 9 4 1 0

35 9 4 1 0

36 9 4 1 0

37 9 4 1 0

38 8 4 1 1

39 9 4 1 1

40 9 4 1 1

41 9 4 1 1

42 9 4 1 1

43 9 4 1 1

Metastasis = bone metastasis determined by whole body scintigraphy or x-ray. The Gleason grading system and the TNM classification (UICC 2002) was used. An exact TNM classification does not exist on patient no. 20 – 27. By androgen deprivation = 1 means that the patient had been castrated prior to surgery.
