# Supplementary material for: Genome-wide analysis of allelic imbalance in prostate cancer using the Affymetrix 50K SNP mapping array
Source: Br J Cancer. 2007 Jan 23;96(3):499–506. doi: 10.1038/sj.bjc.6603476 (PMC2360016; doi:10.1038/sj.bjc.6603476)
Supplement: Supplementary data Table 4 [file 6603476x4.doc]

**Table 4. Supplementary materials**

Comparison of genotype calls samples of matching tumor and germline DNA from prostate cancer patients using Affymetrix GDAS software and SNaPshot single base extension.

SNP Gene No. alleles tested Concordance

rs1864074 LTBP4 22 100%*

rs205351 MAP3K7 24 100%

rs1509478 Serpin B5 22 91%**

rs1116085 PPP3CC 24 100%

rs2449346 SCAM-1 22 100%

* 2/22 showed "No Call" in Affymetrix genotype call, and AB in SNaPshot genotype call

**2/22 was inconclusive by SNaPshot.
