# Supplementary material for: Genome-wide analysis of allelic imbalance in prostate cancer using the Affymetrix 50K SNP mapping array
Source: Br J Cancer. 2007 Jan 23;96(3):499–506. doi: 10.1038/sj.bjc.6603476 (PMC2360016; doi:10.1038/sj.bjc.6603476)
Supplement: Supplementary data Table 5 [file 6603476x5.doc]

**Table 5A. Supplementary materials**

**Genomic regions showing significant alterations in signal intensities in samples of metastatic- as compared to localized prostate cancer.**

**Chr. Start* End* SNPs in Gain/loss Region Size p-value****

**Region (Mb)**

1 201.25 204.51 27 Gain 3.26 0.009

1 208.07 210.66 27 Gain 2.59 0.009

4 10.00 10.43 16 Loss 0.43 0.01

5 34.67 35.19 21 Gain 0.52 0.01

7 42.94 44.99 19 Gain 2.05 0.007

7 128.44 130.40 27 Gain 1.96 0.003

8 95.18 96.72 36 Gain 1.54 0.008

8 123.41 126.82 39 Gain 3.41 0.006

8 133.82 136.26 50 Gain 2.44 0.004

9 74.178 75.08 19 Gain 0.90 0.008

9 125.24 129.79 26 Gain 4.55 0.001

10 68.90 71.20 37 Gain 2.31 0.002

10 72.00 73.93 25 Gain 1.93 0.005

10 94.33 95.10 27 Gain 0.78 0.005

11 65.70 70.86 28 Gain 5.16 0.004

12 102.96 103.60 20 Gain 0.64 0.009

12 107.47 111.44 28 Gain 3.98 0.004

12 115.20 116.42 23 Gain 1.23 0.007

14 20.78 21.54 17 Gain 0.76 0.007

17 70.89 71.76 20 Gain 0.88 0.003

17 74.54 81.25 24 Gain 6.71 0.002

19 16.19 17.53 10 Gain 1.33 0.007

19 34.22 35.19 11 Gain 0.97 0.004

19 50.48 52.29 15 Gain 1.81 0.002

19 53.41 56.01 14 Gain 2.59 0.003

19 59.25 59.89 10 Gain 0.64 0.007

20 2.45 4.19 15 Gain 1.74 0.006

20 23.45 24.10 16 Gain 0.64 0.006

20 42.36 43.87 14 Gain 1.50 0.007

21 38.98 39.16 15 Gain 0.19 0.006

22 45.08 46.44 10 Gain 1.36 0.009

Listed are regions with consecutive SNPs each displaying a difference at 1% significance level in signal intensity of the samples from localized compared to metastatic prostate cancer. Multiple regions at chromosomes 10 and 19 are not necessarily independent regions of altered DNA copy changes, but can be parts of larger regions. False discovery rate of chromosomes with significant segments is less than 1/22=1.5%.

* The genomic positions were based on the May 2004 genome assembly (hg17) of the UCSC human genome browser (<http://genome.ucsc.edu/>).

**** Segments with *p≤* 0.01 were reported

**Table 5B Supplementary materials**

**Genomic regions showing significant alterations in signal intensities between samples of metastatic prostate cancer with and without previous androgen deprivation.**

**Chr. Start* End* SNPs in Gain/Loss Region Size p-value****

**Region (Mb)**

2 42.53 43.91 13 Gain 1.37 0.043

2 53.83 54.09 13 Gain 0.26 0.043

2 55.56 55.88 17 Gain 0.32 0.022

10 60.65 61.33 22 Gain 0.68 0.013

Listed are regions with consecutive SNPs each displaying significant difference (p<0.01) in signal intensity of the samples from metastatic prostate cancer with and without antiandrogen therapy. Androgen deprivation therapy was performed as orchiectomy. Multiple regions at chromosome 2 are not necessarily independent regions of altered DNA copy changes, but can be parts of larger regions.

* The genomic positions was based on the May 2004 genome assembly (hg17) of the UCSC human genome browser (<http://genome.ucsc.edu/>).

**Table 5C Supplementary materials**

**Genomic regions showing significant alterations in signal intensities between samples of Gleason score ≥ 8 as compared to Gleason score ≤7 prostate cancer.**

**Chr. Start* End* SNPs in Gain/Loss Region Size p-value****

**Region (Mb)**

1 143.19 144.80 28 Gain 1.61 0.007

1 196.95 198.04 27 Gain 1.09 0.007

1 198.23 204.51 71 Gain 6.27 0.000

1 204.63 206.06 25 Gain 1.43 0.008

1 209.45 211.35 42 Gain 1.90 0.001

3 156.70 158.44 34 Gain 1.74 0.004

7 127.94 130.31 29 Gain 2.37 0.005

8 70.26 71.75 31 Gain 1.49 0.008

8 80.29 83.07 48 Gain 2.79 0.003

8 94.96 96.44 36 Gain 1.48 0.006

8 98.00 100.48 42 Gain 2.48 0.003

8 100.79 102.90 34 Gain 2.12 0.007

8 109.020 110.62 40 Gain 1.60 0.003

8 122.20 127.94 106 Gain 5.74 0.000

14 32.70 33.98 11 Gain 1.28 0.01

Listed are regions with consecutive SNPs each displaying significant difference (p<0.01) in signal intensity of the samples from low differentiated compared to high differentiated prostate cancer. Multiple regions at chromosomes 1 and 8 are not necessarily independent regions of altered DNA copy changes, but can be parts of larger regions. FDR of chromosomes with significant segments is less than 1/5=20%.

* The genomic positions was based on the May 2004 genome assembly (hg17) of the UCSC human genome browser (<http://genome.ucsc.edu/>).

**** Segments with *p≤* 0.01 were reported.
