# Supplementary figures and images for: Genome-wide analysis of allelic imbalance in prostate cancer using the Affymetrix 50K SNP mapping array
Source: Br J Cancer. 2007 Jan 23;96(3):499–506. doi: 10.1038/sj.bjc.6603476 (PMC2360016; doi:10.1038/sj.bjc.6603476)

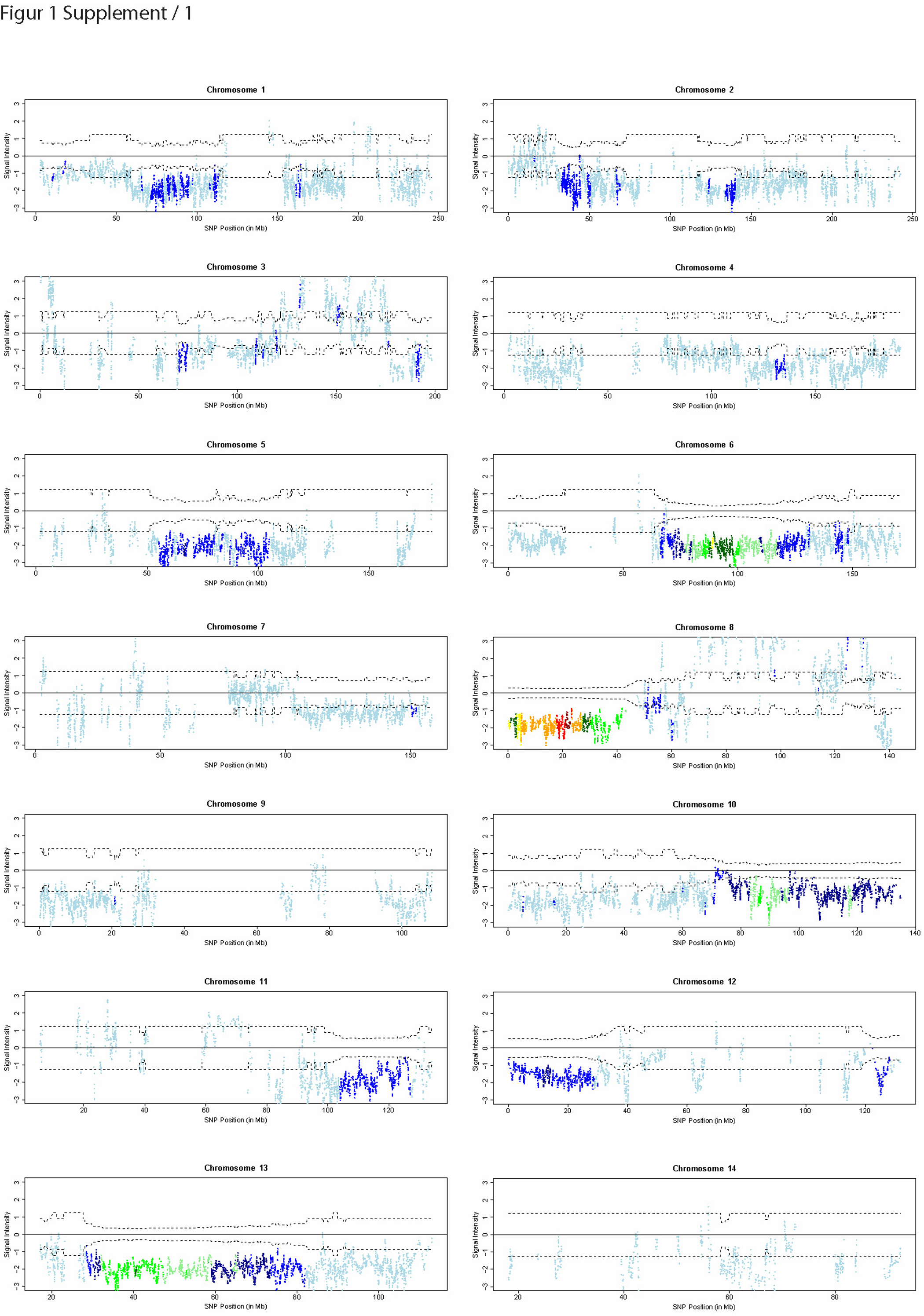

Supplement: Figure 1 Supplement/1 [file 6603476x6.tif]

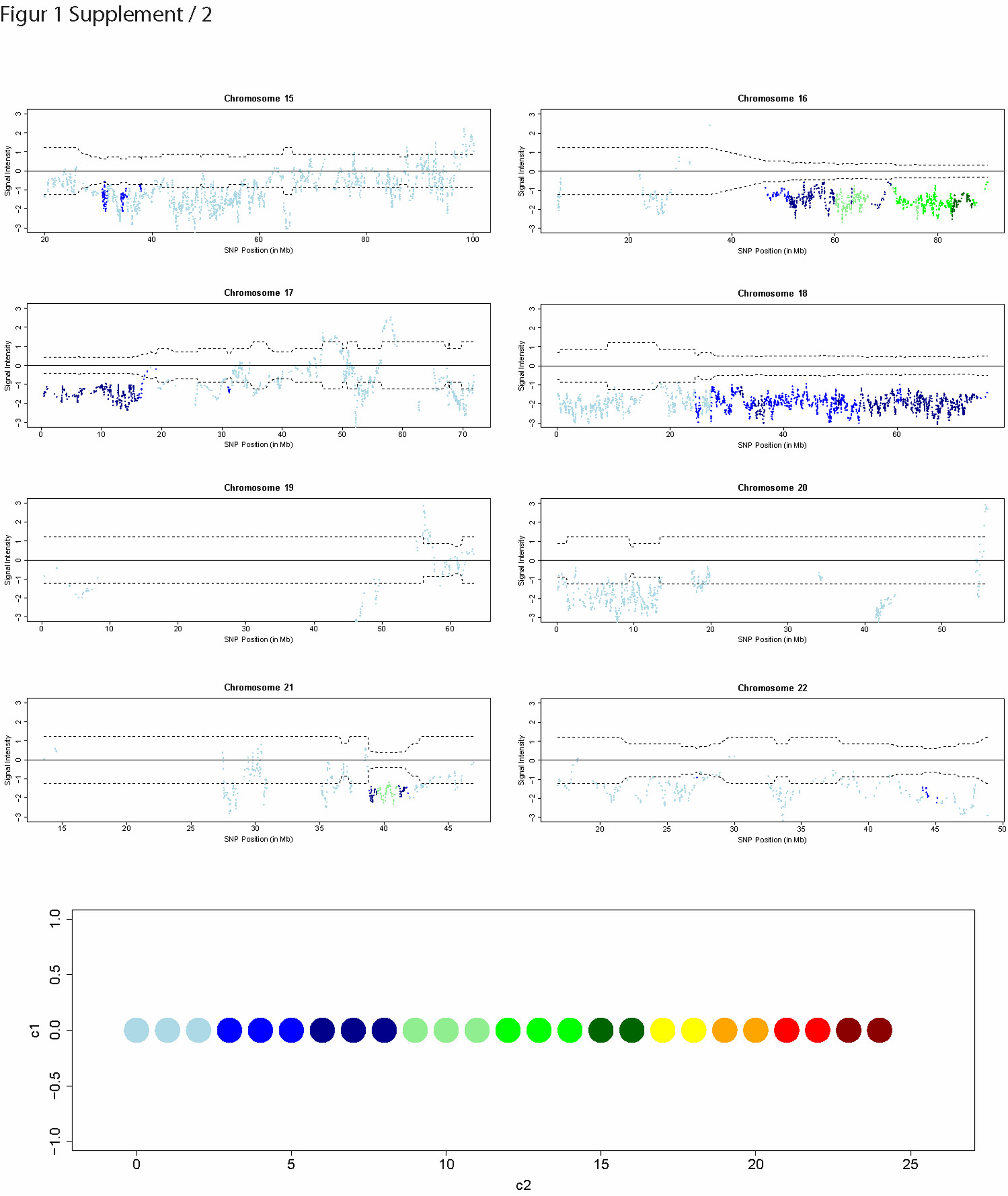

Supplement: Figure 1 Supplement/2 [file 6603476x7.tif]

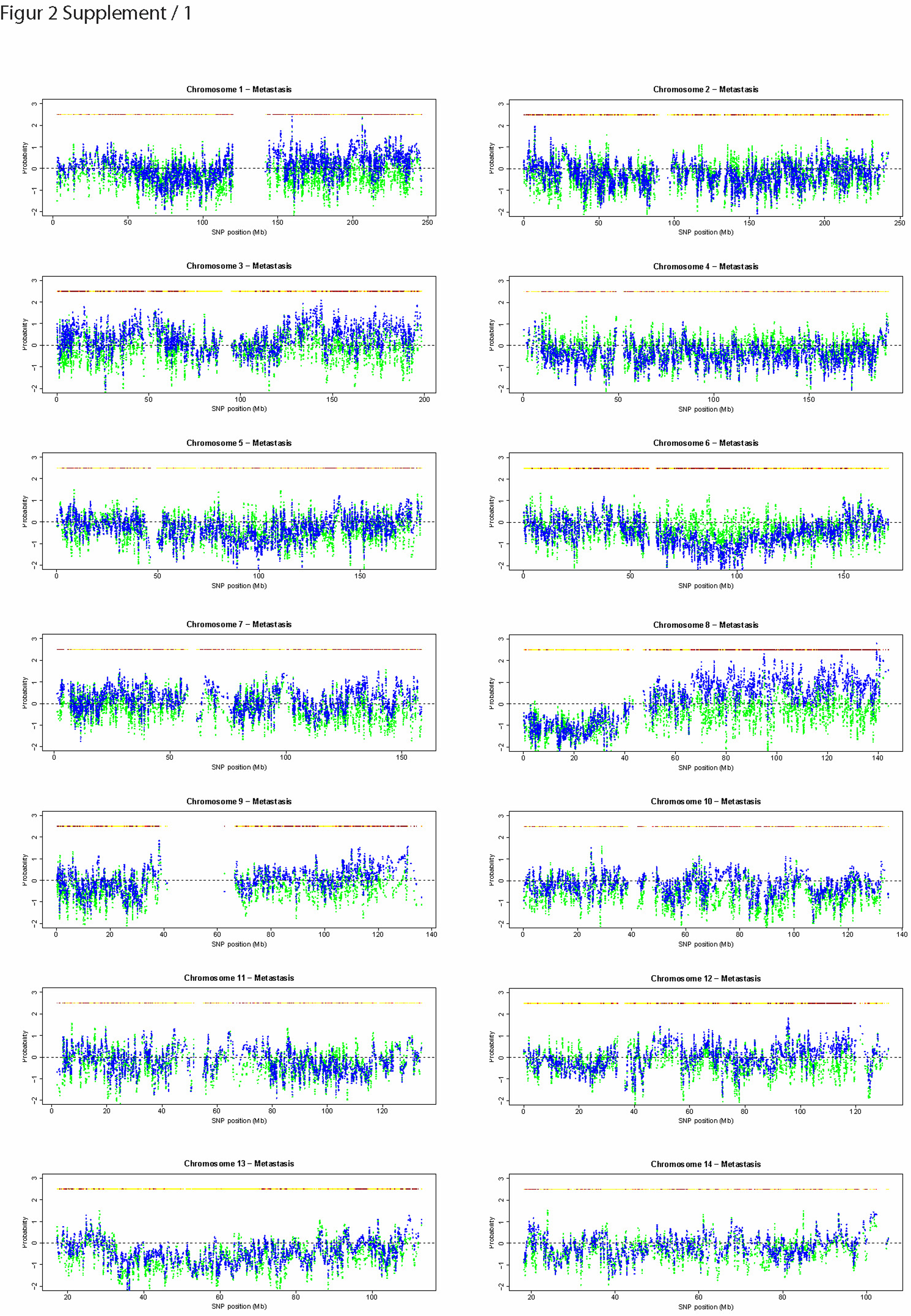

Supplement: Figure 2 Supplement/1 [file 6603476x8.tif]

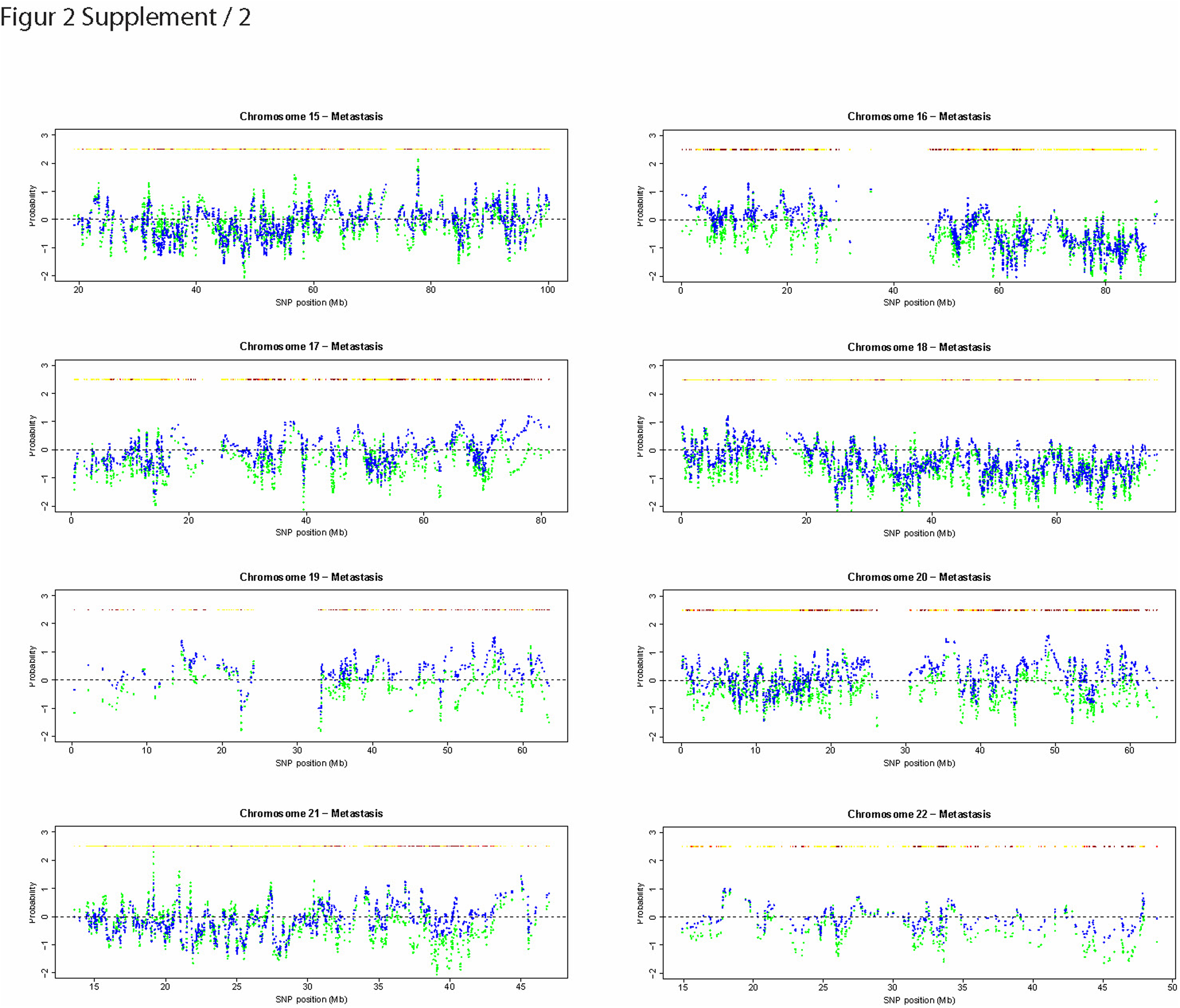

Supplement: Figure 2 Supplement/2 [file 6603476x9.tif]

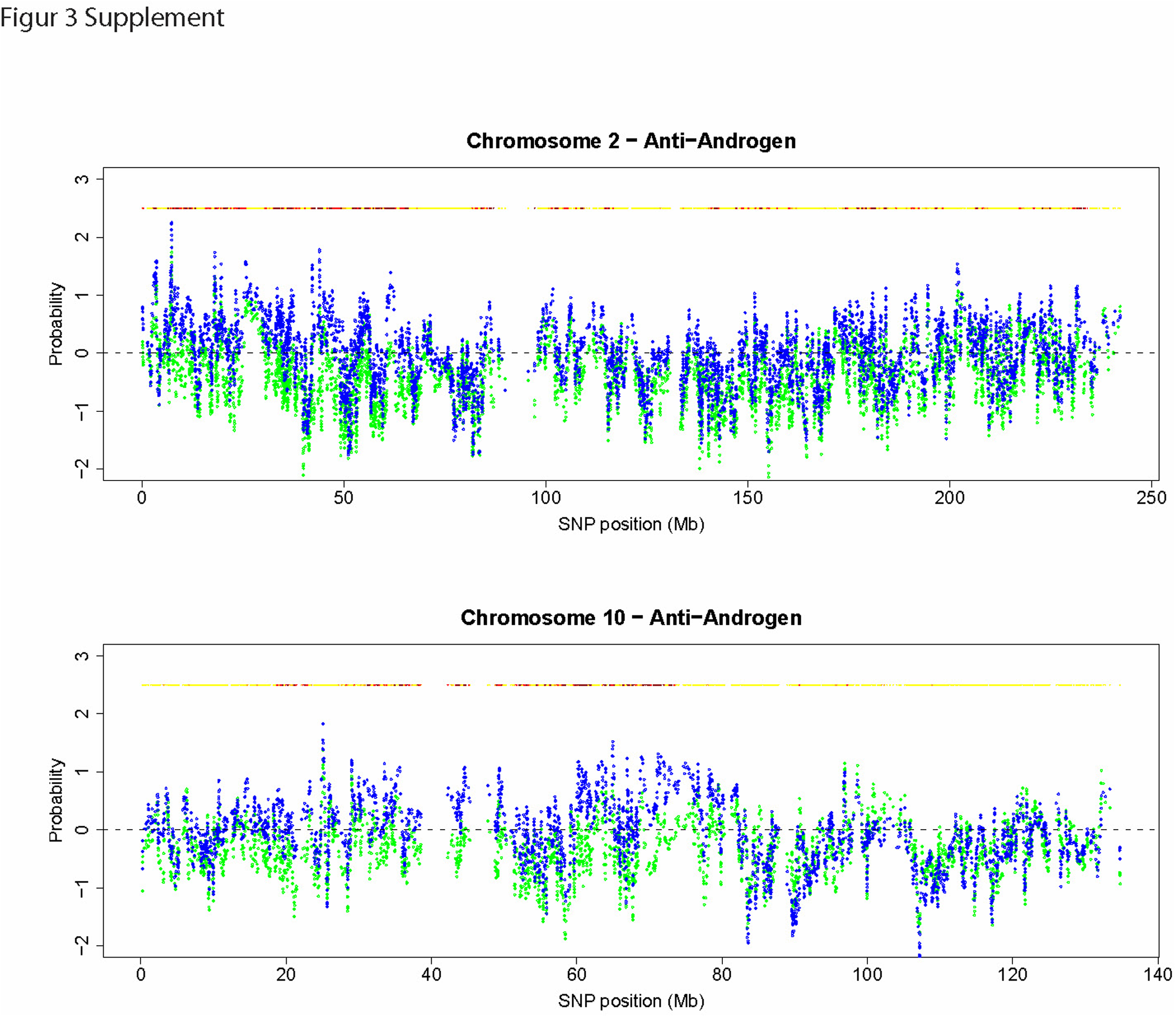

Supplement: Figure 3 Supplement [file 6603476x10.tif]

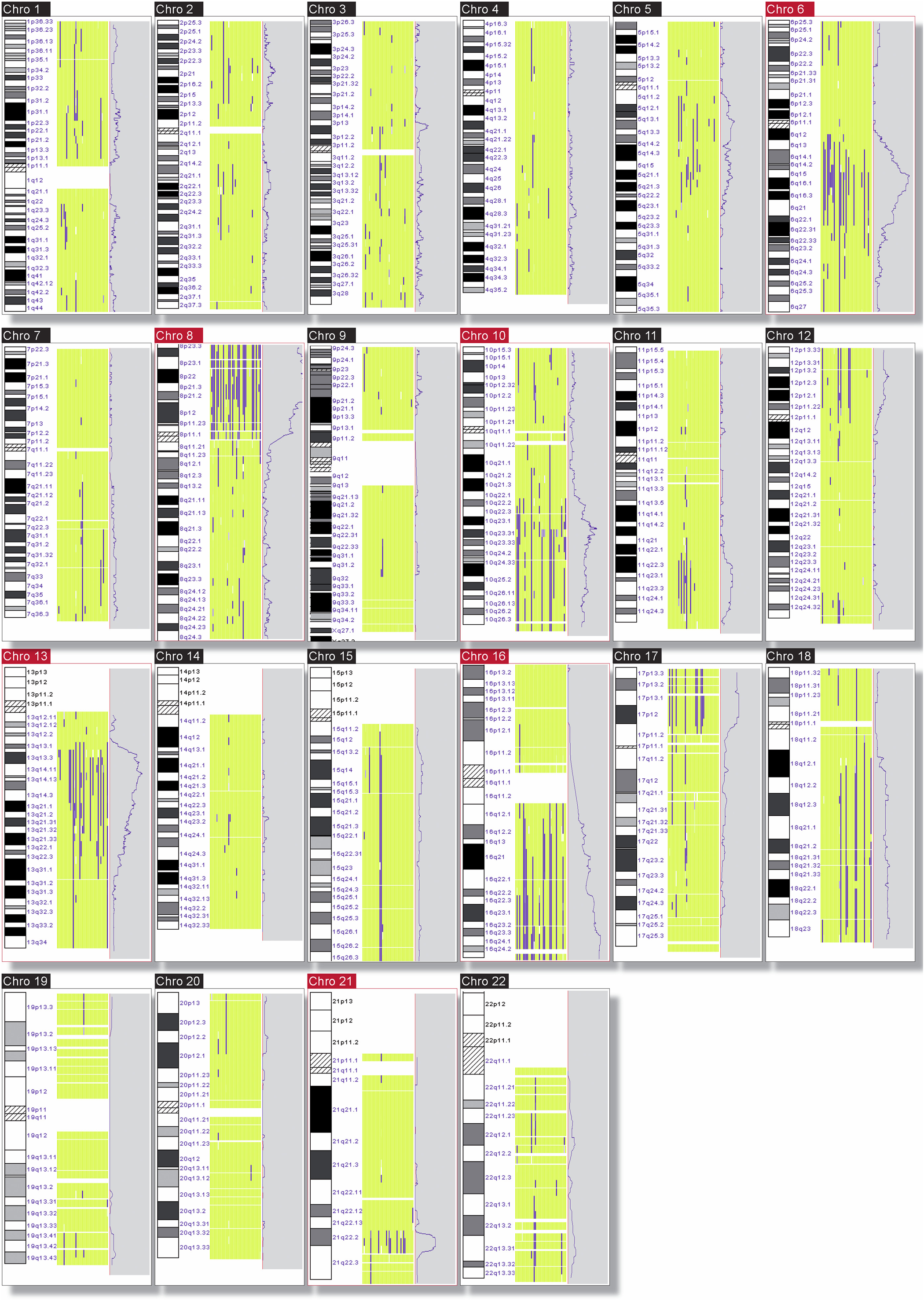

Supplement: Figure 4 Supplement [file 6603476x11.tif]
