# Supplementary material for: Genome-wide analysis of allelic imbalance in prostate cancer using the Affymetrix 50K SNP mapping array
Source: Br J Cancer. 2007 Jan 23;96(3):499–506. doi: 10.1038/sj.bjc.6603476 (PMC2360016; doi:10.1038/sj.bjc.6603476)
Supplement: Legends to Supplementary Figures [file 6603476x12.doc]

**Legends to figures supplementary materials**

**Figure 1**

Correlation of LOH and genomic copy number alterations in samples displaying LOH.

The signal intensity value for a particular SNP was calculated for all tumors with LOH in that SNP and plotted with a colour that indicates the number of tumors with LOH. SNPs with no LOH are left out. Also inserted are the lines corresponding to a significance level of 1% on either side. The widths of these vary because some SNPs experience more LOH than others. SNPs with signal intensities outside the 1% significance level threshold are considered to represent genomic copy numbers different from 2 (if positive >2 and if negative <2). Areas for which LOH and copy number reductions seem to be positively correlated include: 6q, 8p, 10q, 13q, 16q and 21q. Color code showing number of samples with LOH depicted.

**Figure 2**

**Identification of genomic regions positively correlating to metastatic disease in prostate cancer**

Genomic differences between tumor subgroups were identified based on signal intensities.

For each group the signal intensity was calculated and plotted. The significance of the difference of the group means was calculated using a permutation test. Segments of SNPs defined as an uninterrupted series of SNPs all significant at 1% level were found in the distribution obtained by permuting group labels. Group of metastatic disease (blue), localized disease (green). Segments indicated on top: *p≤ 0.01 (brown), 0.01≤ p≤ 0.02 (red); 0.02≤ p≤ 0.05 (orange).*

**Figure 3**

**Identification of genomic regions positively correlating to androgen deprivation therapy in metastatic prostate cancer**

With androgen deprivation (blue); without antiandrogen therapy (green). Segments indicated on top: *p≤ 0.01 (brown), 0.01≤ p≤ 0.02 (red); 0.02≤ p≤ 0.05 (orange).*

**Figure 4**

**Loss of Heterozygosity (LOH) in Chromosomes 1-22 in microdissected prostate cancer tissue as determined by dChip**. LOH regions (blue colour), retained regions (yellow) and uninformative (white) covering the genome in 39 individual samples of matched tumor and germline. Each column represents one tumor/germline pair. Additionally along the right hand side of each figure within the grey shaded box is the average LOH score for the 39 samples. Cytoband for the individual chromosomes is shown on the left hand side.
